# Supplementary material for: Patterns and driving forces of dimensionality-dependent charge density waves in 2H-type transition metal dichalcogenides
Source: Nat Commun. 2020 May 15;11:2406. doi: 10.1038/s41467-020-15715-w (PMC7229047; doi:10.1038/s41467-020-15715-w)
Supplement: Supplementary file 1 — Supplementary Information [file 41467_2020_15715_MOESM1_ESM.pdf]

Supplementary Information: Patterns and driving forces of dimensionality-dependent charge density waves in 2*H*-type transition metal dichalcogenides

Dongjing Lin, Shichao Li, Jinsheng Wen, Helmuth Berger, László Forró, Huibin Zhou, Shuang Jia, Takashi Taniguchi, Kenji Watanabe, Xiaoxiang Xi,* and Mohammad Saeed Bahramy*

*e-mail: xxi@nju.edu.cn; bahramy@ap.t.u-tokyo.ac.jp

Supplementary Note 1. Electronegativity, interlayer charge transfer, and electronic band structure

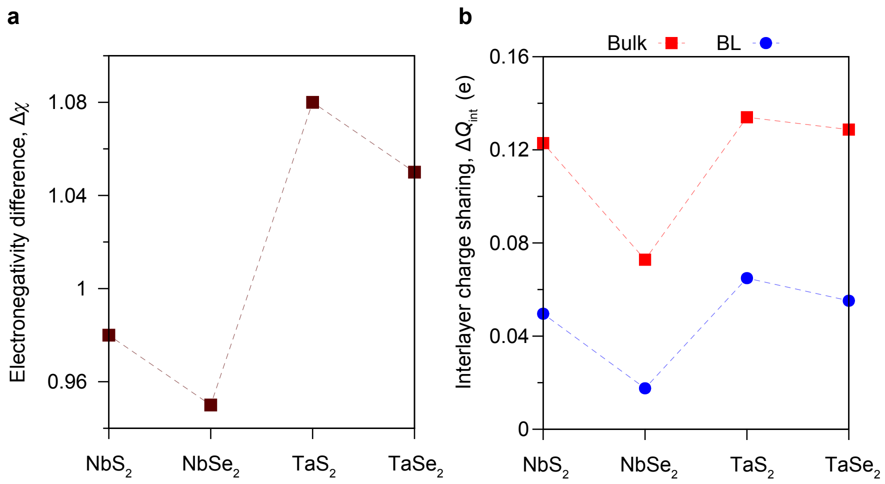

Supplementary Figure 1. **Electronegativity difference and interlayer charge transfer for 2*H*-*MX*$_2$ (*M*=Nb, Ta and *X*=S, Se).** **a** Electronegativity difference $\Delta\chi$ between the transition metal and chalcogen atoms, calculated using the electronegativity values from Supplementary Reference 1. **b** The interlayer charge sharing ΔQ_{int} upon increasing the layer number from a monolayer for bulk and bilayer of all compounds.

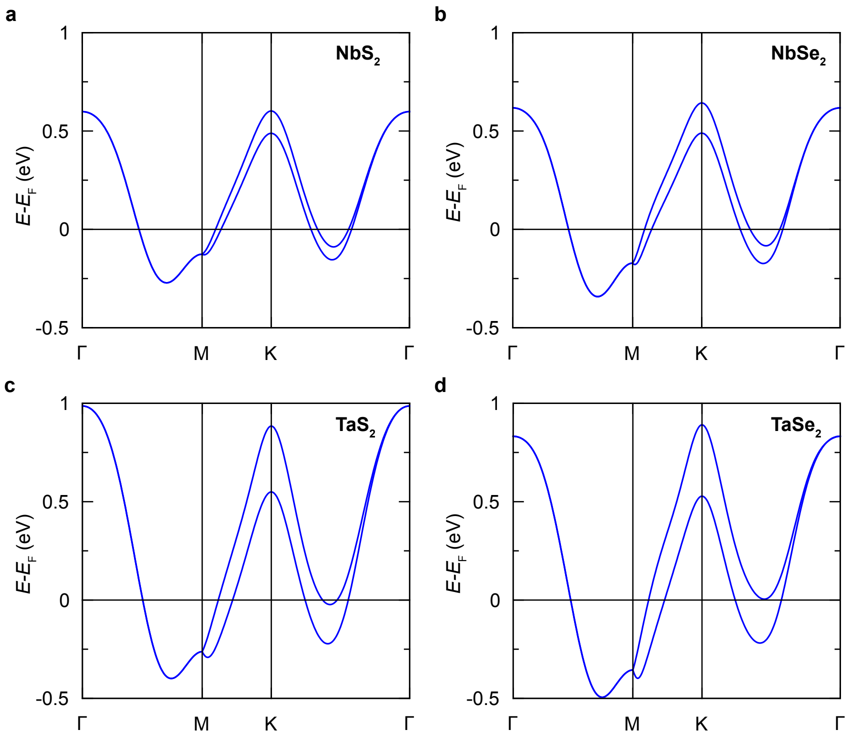

Supplementary Figure 2. **Electronic band structure for monolayer 1*H*-*MX*$_2$ (*M*=Nb, Ta and *X*=S, Se).** The results are obtained from first-principles calculations including spin-orbit coupling.

Supplementary Note 2. Raw data for Raman scattering

Supplementary Figure 3 shows the raw Raman scattering data for all samples measured. We can make the following observations based on mere visual inspection of the data. In all samples the A_{1g} and E_{2g} phonon peaks blueshift upon decreasing temperature. We discuss other features in the data in the order of descending sample thickness, i.e. from the bulk to the monolayer.

- $NbSe_2$. The amplitude mode at base temperature blueshifts, is strongly enhanced, and extends to higher temperatures. The two-phonon scattering peak becomes weaker but shows a similar temperature dependence. The zone-folded mode at $\sim$190 cm^{-1} becomes more pronounced.
- $TaSe_2$. The amplitude modes are at similar energies and span a similar temperature range. The two-phonon scattering peak is significantly weaker. The zone-folded modes near 200 cm^{-1} are also weaker.
- TaS_2. The general features of the amplitude modes and the two-phonon peaks are very similar to those in $TaSe_2$, except that the monolayer shows strongly suppressed amplitude modes. A weak zone-folded mode is observed in the bulk near 100 cm^{-1}.
- NbS_2. No amplitude modes or zone-folded modes are observed from the bulk down to the monolayer. The two-phonon scattering peaks near 250 cm^{-1} are observed in all samples, and the bilayer and monolayer show extra broad peaks bellow 200 cm^{-1}.

Detailed analysis of all these features can be found in Supplementary Note 3–10.

Supplementary Note 3. Layer number dependence of the phonon mode frequency

The first-order phonon scattering peaks in $2H$-MX_2 (M=Nb, Ta and X=S, Se) show characteristic layer number dependences, providing a convenient method to determine the layer number. This has been well established for $NbSe_2$[2] as well as in other similar layered materials such as $2H$-MoS_2[3]. Supplementary Figure 4 shows the layer number dependence of the phonon frequencies for the shear mode, A_{1g}, and E_{2g} modes. The mode frequencies are extracted by performing least-squares fits of the phonon peaks using a Lorentzian function, except for the E_{2g} mode of TaS_2, for which a Fano lineshape is required (see Supplementary Note 5). Upon reducing the layer number, the former two decrease while the latter increases. The shear mode frequencies, when normalized to the corresponding values of the bulk sample, collapse onto a single curve, which can be empirically parametrized as $\omega(N)/\omega(\text{bulk}) = \cos(\pi/2N)$, where N is the layer number. It is reduced by $\sim$30% when going from the bulk to the bilayer. The absence of the shear mode can be taken as a definitive signature for a monolayer.

Supplementary Note 4. Temperature dependence of the phonon mode frequency

Phonon-phonon interaction (known as the anharmonic effect) renormalizes phonon frequency and linewidth. Because phonon occupation obeys Bose-Einstein statistics,

$$n(\omega, T) = \frac{1}{e^{\hbar\omega/k_B T} - 1}, \tag{1}$$

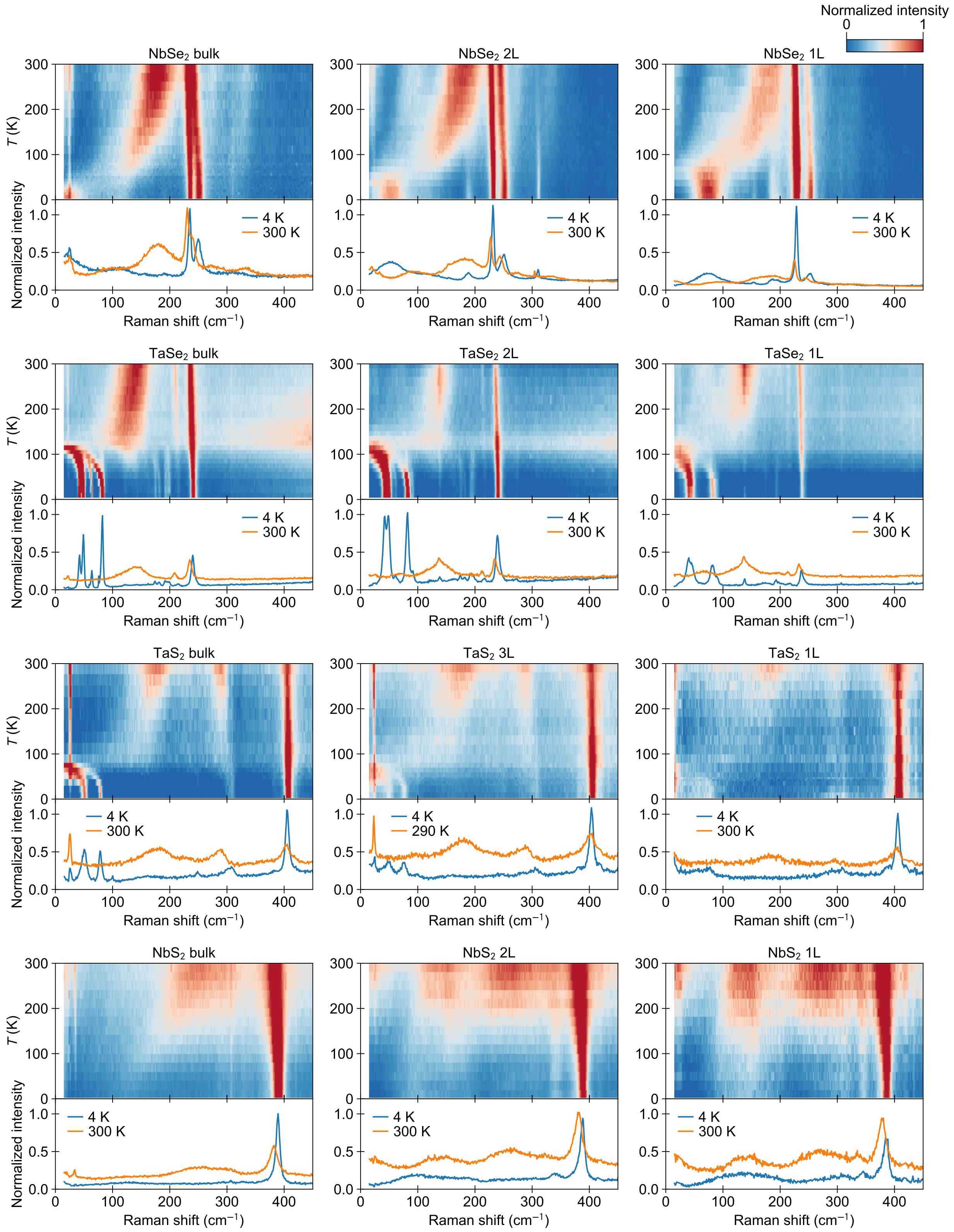

Supplementary Figure 3. Temperature and thickness dependence of the raw Raman scattering data for 2H-MX_2 (M=Nb, Ta and X=S, Se). The upper part of each panel shows a Raman scattering intensity map, and the lower part compares the spectra at 4 K and 300 K. All data were collected in the parallel polarization configuration. Data for each sample are scaled by a constant to allow for comparison among different samples. All data share the same colour scale.

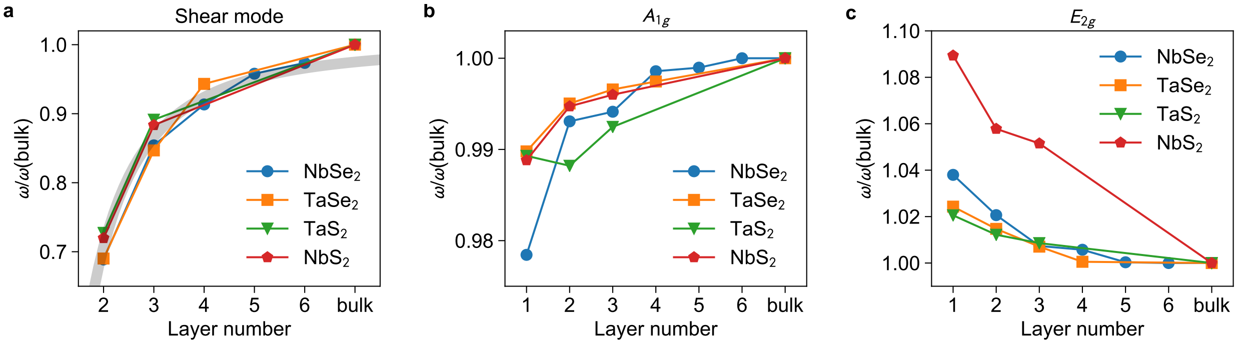

Supplementary Figure 4. **Determination of the layer number.** Layer-number dependence of the phonon frequencies for **a** shear mode, **b** A_{1g} mode, and **c** E_{2g} mode. All data are normalized to the values of the corresponding bulk samples. The thick grey line in **a** shows a $\cos(\pi/2N)$ dependence on the layer number N.

where ω is the phonon frequency and k_B the Boltzmann constant, the renormalized phonon frequency and linewidth are both expected to show temperature dependence. The theoretical explanation for this effect was originally developed by Cowley and Klemens and later adapted to interpret various experiments[4,5]. Here we adopt the following simplified expressions for data analysis. Define ω_0 as the phonon frequency in the harmonic model. The renormalized phonon frequency is

$$\omega(T) = \omega_0 + A[1 + 2n(\omega_0/2, T)] \tag{2}$$

or

$$\omega(T) = \omega_0 + A[1 + 2n(\omega_0/2, T)] + B[1 + 3n(\omega_0/3, T) + 3n(\omega_0/3, T)^2]. \tag{3}$$

The former assumes anharmonic decay of the phonon with energy $\hbar\omega_0$ into two LA phonons of equal energy, while the latter also considers the decay into three phonons of equal energy.

Supplementary Figure 5 shows the temperature dependence of the A_{1g} mode frequency for all samples. Supplementary Equation 2 fits the data reasonably well. The magnitude of the constant A, i.e. $|A|$, can be taken as a means to characterize the degree of the anharmonic effect. Interestingly, Supplementary Figure 5f shows that $NbSe_2$, $TaSe_2$, and TaS_2 have similar $|A|$, while the values for NbS_2 samples of different thickness are all about three times higher. This is consistent with the proposal that the strong lattice anharmonicity in bulk NbS_2 is the cause for the absence of CDW[6,7]. Bilayer and monolayer NbS_2 both have a similar value of $|A|$ as that of the bulk, indicating that the level of lattice anharmonicity remains mostly unchanged when approaching the monolayer limit. This is consistent with our observation of the absence of CDW in this system (see Supplementary Note 2). For comparison, we also show the temperature dependence of the A_{1g} mode frequency of bilayer MoS_2 in Supplementary Figure 5e (measured using 532 nm excitation), together with the fitting results using Supplementary Equation 2. Its $|A|$ value, shown in Supplementary Figure 5f, is close to those of $TaSe_2$.

The E_{2g} modes are much weaker than the A_{1g} modes, making quantitative analysis more challenging. For completeness, in Supplementary Figure 6 we include those results with reasonable quality. While the data for monolayer NbS_2 and bilayer MoS_2 can be fitted well using Supplementary Equation 2, those for $NbSe_2$ and $TaSe_2$ require Supplementary Equation 3 to properly account for the upturn at high temperature. We are therefore not able to make a systematic comparison of the degree of lattice anharmonicity for the E_{2g}

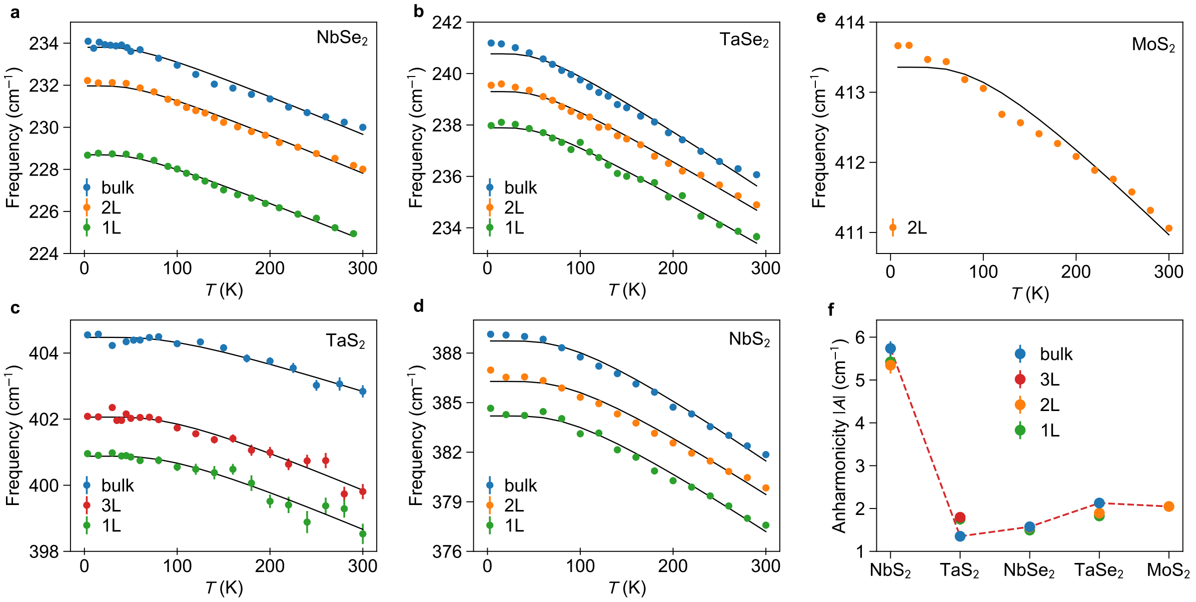

Supplementary Figure 5. Temperature dependence of the A_{1g} phonon frequency. Error bars for the frequencies are the standard deviations derived from the least-squares peak fitting analysis, as detailed in Supplementary Note 3. The solid lines in **a–e** are fits using Supplementary Equation 2, with the magnitude of one of the fitting parameters $|A|$ and associated error bars plotted in **f**.

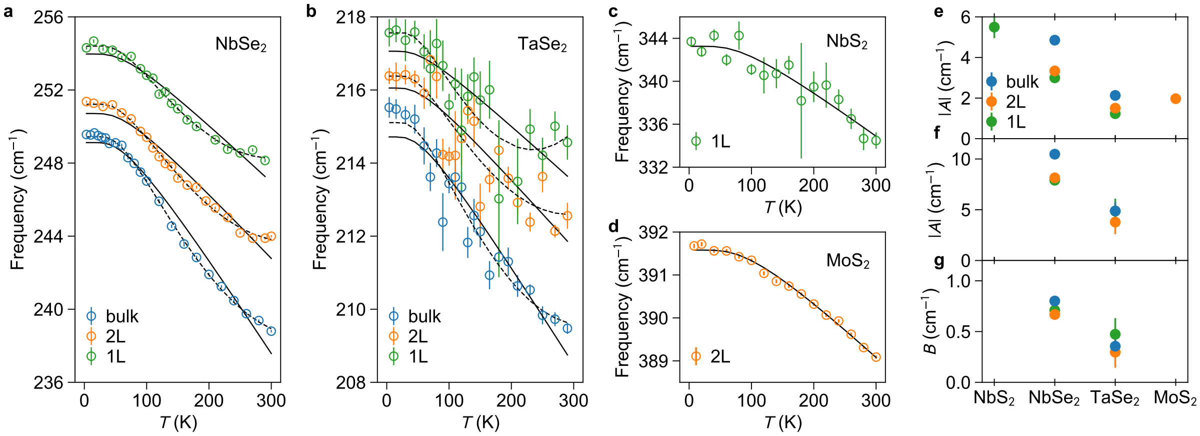

Supplementary Figure 6. Temperature dependence of the E_{2g} phonon frequency. The solid lines in **a–d** are fits using Supplementary Equation 2, with $|A|$ plotted in **e**. The dashed lines in **a–b** are fits using Supplementary Equation 3, with $|A|$ and B plotted in **f** and **g**, respectively. Origins for the error bars are the same as those explained in Supplementary Figure 5.

modes among different materials. Supplementary Figure 6e–g roughly sort NbS_2, $NbSe_2$, and $TaSe_2$ in the order of decreasing lattice anhamonicity. The value of $|A|$ for bilayer MoS_2 is again similar to that of $TaSe_2$.

Supplementary Note 5. Fano lineshape for the E_{2g} mode in TaS_2

The lineshape of the E_{2g} mode in TaS_2 shows clear asymmetry, which can be quantitatively described by the Fano theory[8], $I(\omega) = A\left[q\gamma + (\omega - \omega_0)\right]^2 / [\gamma^2 + (\omega - \omega_0)^2]$. Here ω_0 is the phonon frequency, γ is the linewidth, and A is constant. q is a dimensionless Fano parameter to characterize the degree of asymmetry. In the limit $|q| \to \infty$ the Lorentzian lineshape is recovered. Supplementary Figure 7a–b show the 300 K Raman spectra of bulk TaS_2 collected in the collinear and cross polarization configurations. We adopt a model consisting of a linear background term, a Gaussian term for the two-phonon peak, a Fano function for the E_{2g} mode, and an extra Lorentzian term for the A_{1g} mode in the collinear polarization configuration. The resultant fits, shown as the solid lines through the data, are quite good. However, replacing the Fano function by a Lorentzian function clearly degrades the quality of the fits (dotted lines). The analysis strongly indicates the presence of Fano resonance. The origin for the Fano resonance requires further investigation.

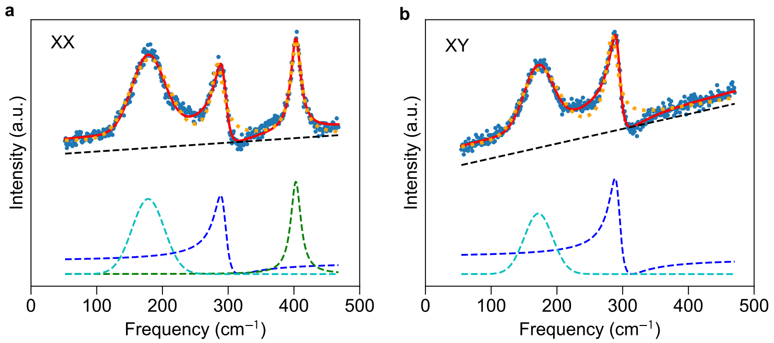

Supplementary Figure 7. The E_{2g} mode in TaS_2. Raman data for bulk TaS_2 collected in the **a** collinear and **b** cross polarization configurations at 300 K. The solid lines are fits using the components shown as dashed lines (a linear background, a Gaussian function for the two-phonon peak, a Fano function for the E_{2g} mode, and a Lorentzian function for the A_{1g} mode). The dotted lines are fits using a Lorentzian function for the E_{2g} mode.

Supplementary Note 6. Analysis of the amplitude mode intensity

In Figure 4 of the main text, we show the normalized Raman spectra, $I/I_0 - 1$, to ease comparison among different samples. Here I is the raw spectra and I_0 is a high-temperature spectrum far above $T_{\rm CDW}$. This temperature was chosen as 64 K for bulk $NbSe_2$ and above 190 K for all other samples. We estimate the integrated intensity for the amplitude modes using the following method. A liner fit is performed on the featureless background surrounding the amplitude modes. We then integrate the amplitude mode intensity with respect to this background. Representative analysis procedures for the bulks samples are demonstrated in Supplementary Figure 8, with the dashed lines showing the linear fits and the shaded areas illustrating the integrated intensity. For $TaSe_2$, some of the low-energy peaks do not shift with temperature (see Supplementary Figure 10e–g) and therefore cannot be assigned as amplitude modes. We do not distinguish these modes here but rather lump them into the integration for simplicity. This is justified for the purpose of estimating $T_{\rm CDW}$ because they are also CDW-induced modes, feature strongly temperature-dependent intensities, and disappear above $T_{\rm CDW}$.

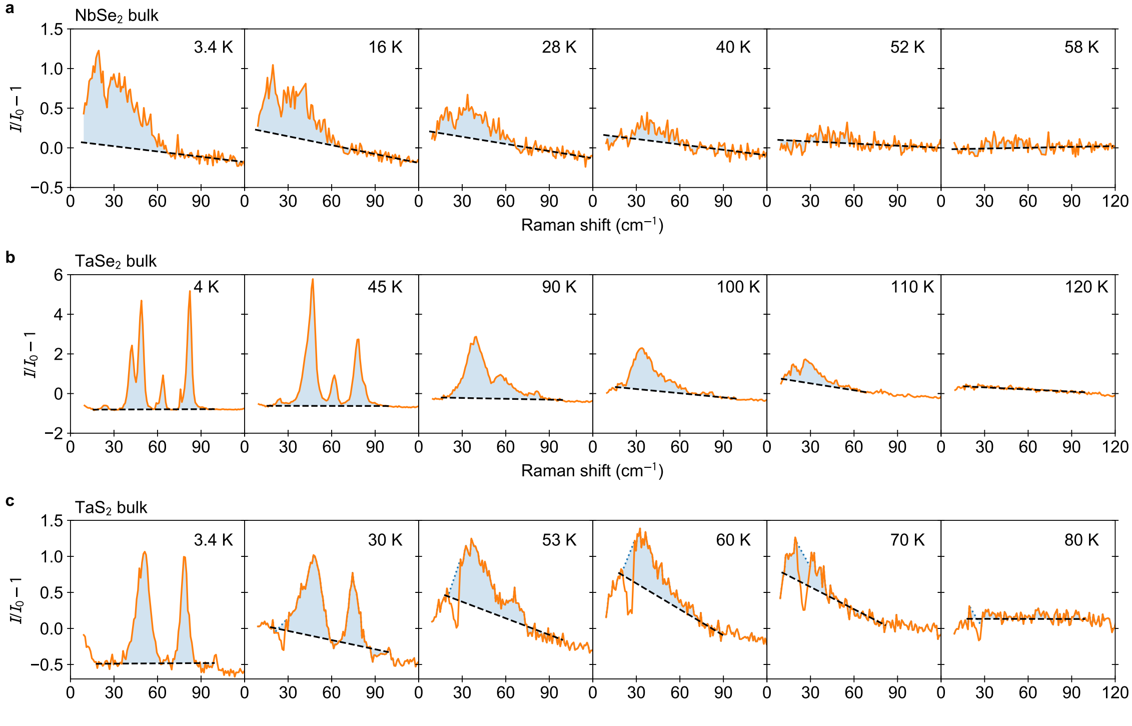

Supplementary Figure 8. **Obtaining the integrated intensity of the amplitude modes.** The featureless region of each $I/I_0 - 1$ spectrum is linearly fitted to yield a background (dashed lines). The intensity of the CDW-induced modes is then integrated with respect to this background, as indicated by the shaded regions. For spectra strongly affected by the shear modes, we patch over the corresponding region by linear interpolation (shown by the dotted lines) before integration. Demonstrations of this method are shown for bulk samples of **a** NbSe$_2$, **b** TaSe$_2$, and **c** TaS$_2$.

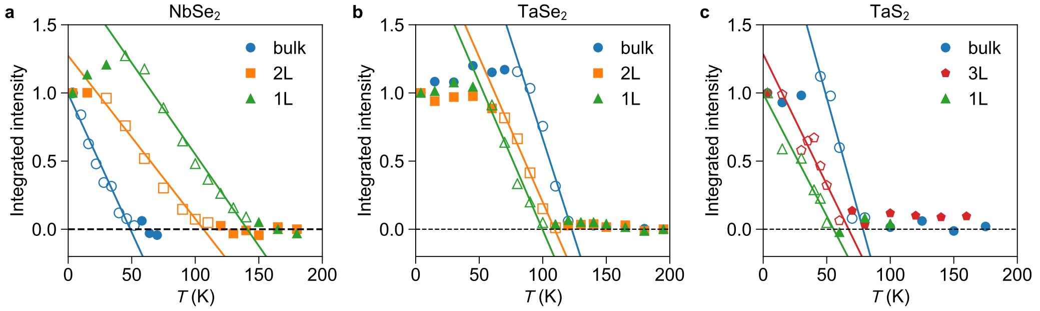

Supplementary Figure 9. **Extracting** T_{CDW} **from the integrated intensity of the amplitude modes.** The sharply-varying part of the temperature-dependent integrated intensity (open symbols) is fitted to a linear function with slope a, $a(T - T_{\mathrm{CDW}})$, by the least-squares method, with a and T_{CDW} being fitting parameters. Error bars of T_{CDW} are also derived from the fit.

Supplementary Figure 9 shows the temperature dependence of the integrated amplitude mode intensity for NbSe$_2$, TaSe$_2$, and TaS$_2$ samples of differing thickness. To estimate T_{CDW}, a linear fit is performed on the sharply-varying part of the data sets (open symbols). The extracted transition temperatures are listed in Supplementary Table 1 and plotted in Supplementary Figure 12 as filled circles.

Supplementary Note 7. Analysis of the amplitude mode frequency

Within the mean field theory for the Peierls transition[9], the amplitude mode frequency exhibits a mean-field type temperature dependence,

$$\omega_{\mathrm{A}}(T) \propto \lambda^{1/2}\Omega_0\sqrt{1-\frac{T}{T_{\mathrm{CDW}}}}, \tag{4}$$

where λ is the electron-phonon coupling constant and Ω_0 is the frequency of the unscreened longitudinal acoustic (LA) phonon of the pristine atomic chain at the CDW wavevector. With this in mind, we perform fitting analysis to extract the amplitude mode frequency as a function of temperature. The results are shown in Supplementary Figure 10. In our fitting model we assume a linear background term, upon which one or several Lorentzian terms are superimposed depending on the specific situation. Example fits are shown as smooth solid lines for $NbSe_2$ and TaS_2.

Bulk $TaSe_2$ exhibits three peaks which redshift upon increasing temperature, all qualitatively consistent with the property of amplitude modes. In the bilayer and monolayer, two of the three peaks remain at approximately the same energies as those in the bulk, while the weakest one near 63 cm^{-1} in the bulk redshifts slightly to 60 cm^{-1} in the bilayer and becomes no longer observable in the monolayer. We are however not able to fit the temperature dependence of these modes according to Supplementary Equation 4. Instead we modify the equation to include an exponent α as one of the fitting parameters[10],

$$\omega_{\mathrm{A}}(T) = \omega_{\mathrm{A}}^0\left(1-\frac{T}{T_{\mathrm{CDW}}}\right)^{\alpha}, \tag{5}$$

which gives reasonable fits as shown in Supplementary Figure 10h. We obtained $\alpha = 0.16 \pm 0.01$, 0.16 ± 0.01, and 0.17 ± 0.02 for the bulk, bilayer and monolayer samples. The extracted T_{CDW} is highly dependent on the last available mode frequency close to the transition, hence only sets a lower bound for the real transition temperature. We also note the existence of a mode near 40 cm^{-1} that does not show appreciable temperature dependence for its frequency in samples of all thickness, indicated by the dashed lines in Supplementary Figure 10e–g. These modes weaken upon increasing temperature, reminiscent of the zone-folded modes, as will be discussed in Supplementary Note 8.

TaS_2 shows a similar temperature dependence for its amplitude mode frequencies as that of $TaSe_2$. For the bulk and bilayer, two amplitude modes appear at approximately the same frequencies. In the monolayer only the higher energy peak can be clearly resolved due to the poor signal-to-noise ratio of the data. We obtained $\alpha = 0.23 \pm 0.02$, 0.05 ± 0.01, and 0.04 ± 0.02 for the bulk, trilayer and monolayer samples, respectively.

$NbSe_2$ exhibits completely different behaviors for the amplitude modes. Only a single broad peak is observed for samples of differing thickness. The mode frequency more than triples when approaching the monolayer limit. In the bulk and bilayer samples, we observe clear redshift for the mode frequency upon increasing temperature, while in the monolayer only a modest reduction of 3.4% is seen from 4 K up to 75 K. Determination of the mode frequency at higher temperatures are unreliable, mostly due to the influence from the adjacent two-phonon scattering peak. From Supplementary Figure 10d one can infer a strongly enhanced T_{CDW} when approaching the monolayer limit.

The extracted transition temperatures for $TaSe_2$ and TaS_2 are listed in Supplementary Table 1 and plotted in Supplementary Figure 12 as open squares.

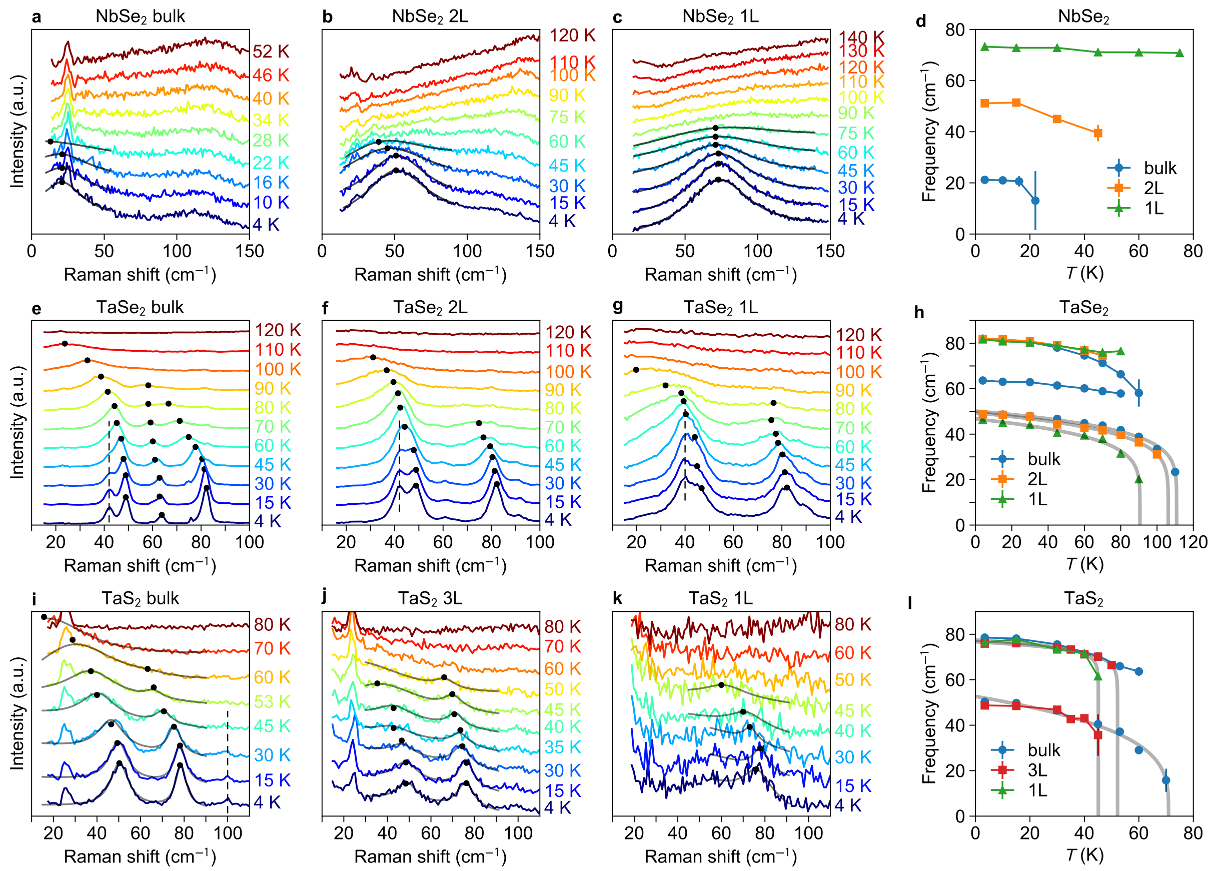

Supplementary Figure 10. **Analysis of the amplitude mode frequency.** The first three columns show temperature-dependent Raman scattering spectra for $NbSe_2$, $TaSe_2$, and TaS_2 samples of various thickness, corresponding to those in Supplementary Figure 3. Data are displaced vertically for clarity. The smooth solid lines are fits using a model combining a linear background and Lorentzian functions. The dots mark the peak frequency of the amplitude modes as obtained from the fits, the values of which are shown in the last column. Error bars in the last column represent the uncertainties associated with the peak fitting analysis. Some of the results for $TaSe_2$ and TaS_2 in the last column are fitted using Supplementary Equation 5 (thick grey lines). The vertical dashed lines in **e**–**g** and **i** delineate another peak assigned as a zone-folded mode.

Supplementary Note 8. Analysis of the zone-folded modes

The CDWs in the materials of our study are either commensurate or near-commensurate with the underlying lattice. This leads to zone folding in the momentum space, which could fold zone-boundary phonon branches back to the Γ point and render some of them Raman active. These zone-folded modes are only expected in the CDW phase, serving as another signature for the CDW transition. We indeed observe such modes in $NbSe_2$[11] and $TaSe_2$[12] samples of all thickness (Supplementary Figure 11) as well as in bulk TaS_2[10] (Supplementary Figure 10i). One key feature of the zone-folded modes is the insensitivity of their frequency to temperature variation. Remarkably, we observe zone-folded modes at nearly the same frequencies in samples of differing thickness for $NbSe_2$ and $TaSe_2$. In TaS_2 the zone-folded mode is only seen in the bulk[10] and becomes no longer observable in atomically thin samples, probably due to degraded signal-to-noise ratio.

A single peak located near 190 cm^{-1} is observed in $NbSe_2$, which becomes more pronounced and persists to much higher temperature in the monolayer, attesting to the enhanced T_{CDW}. Fits based on a combination of a linear or quadratic term for the background and a Lorentzian term for the zone-folded mode allow for the extraction of the mode area, as plotted in Supplementary Figure 11d. We estimate T_{CDW} by linear fits of all or part of the data in Supplementary Figure 11d. The obtained values indeed confirm enhanced T_{CDW} in atomically thin samples.

In $TaSe_2$ multiple peaks are observed between 160–210 cm^{-1}, all of which weaken upon increasing temperature. At 110 K these modes are still discernible in the bulk but not as clear in the bilayer. Likewise, at 100 K these modes are easily seen in the bilayer but has faded away in the monolayer. Therefore T_{CDW} monotonically decreases when approaching the monolayer limit, opposite to the case of $NbSe_2$. Since the peaks strongly overlap, we resort to simple spectral integration rather than fitting analysis to obtain the integrated area. The results are shown in Supplementary Figure 11h. T_{CDW} is again estimated by linear fits to part of the data in Supplementary Figure 11h.

The obtained T_{CDW} values for $NbSe_2$ and $TaSe_2$ samples of various thickness are included in Supplementary Table 1 and plotted in Supplementary Figure 12 as open triangles.

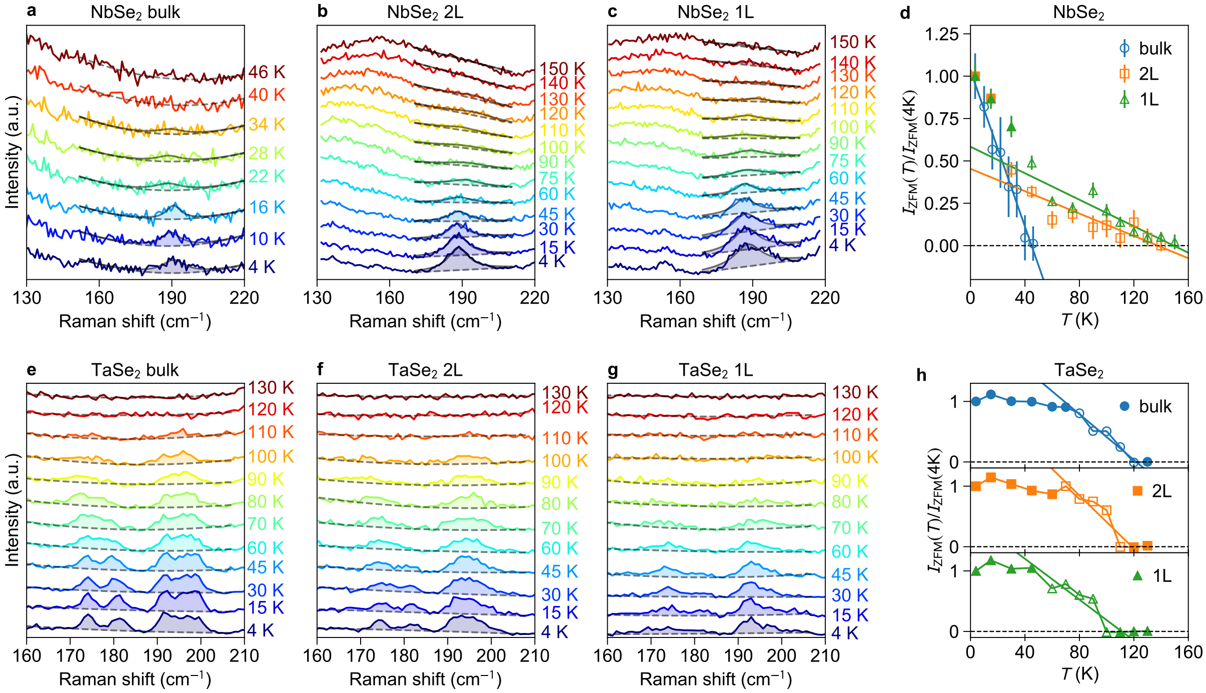

Supplementary Figure 11. **Analysis of the zone-folded modes. a–c** Raman scattering spectra for $NbSe_2$ corresponding to those in Supplementary Figure 3, vertically shifted for clarity. The spectral range spanning the zone-folded mode is fitted to a model combining either a quadratic or a linear background term (dashed lines) and a Lorentzian function. The resultant fits are shown as smooth solid lines. The shaded regions indicate the integrated intensity, whose temperature dependence is summarized in **d** together with linear fits to estimate T_{CDW}. Error bars in **d** represent the uncertainties associated with the peak fitting analysis. The second row shows the results for $TaSe_2$. A direct spectral integration is employed rather than following a fitting procedure.

Supplementary Note 9. Summary of T_{CDW} analyzed using different methods

Here we summarize the T_{CDW} of all samples analyzed using different methods. The results are listed in Supplementary Table 1 and plotted in Supplementary Figure 12 for comparison. Method 1, 2, and 3 are based on the analysis of the amplitude mode intensity (Supplementary Note 6), the amplitude mode frequency (Supplementary Note 7), and the zone-folded mode intensity (Supplementary Note 8), respectively. The errors are given by the least-squares fitting procedures detailed in each section. The trend of the layer number dependence of T_{CDW} for each compound is highly consistent among different methods, although the specific values depend on the choice of method.

Supplementary Table 1. Summary of T_{CDW} obtained from different methods. Each method is described in the text.

Material		T_{CDW} (K)		
		Method 1	Method 2	Method 3
$NbSe_2$	bulk	48.9 ± 4.2	–	44.9 ± 3.9
	2L	106.4 ± 9.8	–	137.5 ± 29.9
	1L	140.9 ± 8.2	–	149.3 ± 28.1
$TaSe_2$	bulk	122.8 ± 16.2	110.8 ± 0.3	121.8 ± 21.6
	2L	109.3 ± 8.6	106.2 ± 0.2	118.6 ± 38.7
	1L	99.7 ± 13.1	90.6 ± 0.2	111.1 ± 49.3
TaS_2	bulk	78.5 ± 17.1	70.4 ± 0.2	–
	3L	67.2 ± 11.4	53.2 ± 1.8	–
	1L	55.4 ± 7.9	45.1 ± 0.2	–

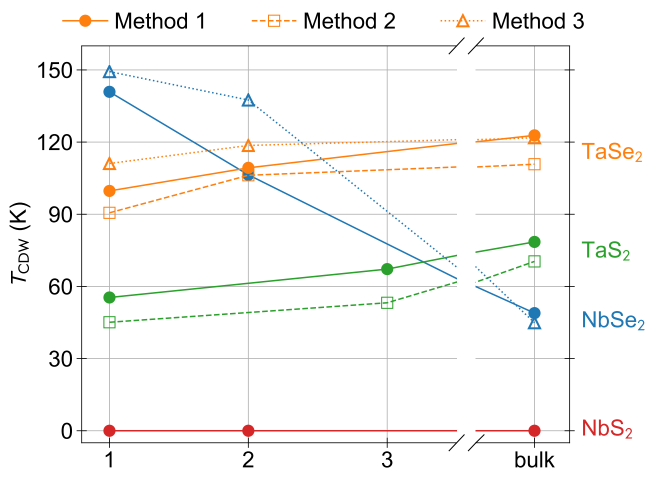

Supplementary Figure 12. Comparison of T_{CDW} obtained using different methods. Each method is described in the text. Different compounds are distinguished by different colors.

Supplementary Note 10. Analysis of the second-order phonon modes

All the samples we study here share a common broad feature at room temperature, which are well known in the literature as due to a two-phonon scattering process[10,11,13]. Noting that at room temperature the energy of this peak is roughly twice that of the Kohn anomaly in the LA phonon dispersion and that it clearly softens upon reducing temperature, this second-order scattering is interpreted as due to two such phonons with opposite momentum. The large scattering cross section for this two-phonon peak, comparable to the first-order phonon peaks, also indicates its unusual origin. Theory shows that the amplitude of this two-phonon scattering is proportional to $|\pi(\mathbf{q})|^2$, where π is the amount of renormalization of the phonon self energy due to electron-phonon interaction and $\mathbf{q}$ is the wavevector for the LA phonon Kohn anomaly[14]. Therefore the intense two-phonon peaks observed in these materials are taken as evidence for strong electron-phonon coupling.

The bulk samples of all compounds exhibit significant variation of the two-phonon peak upon cooling, as shown in the first column of Supplementary Figure 13. For quantitative analysis, we fit the two-phonon peaks using a combination of a linear term and a Gaussian term. The peak frequency thus determined are plotted in the last column of Supplementary Figure 13. For bulk $NbSe_2$, the mode frequency keeps declining down to 4 K, while in bulk $TaSe_2$ and TaS_2 it turns around at T_{CDW}, showing a minimum that signifies the CDW transition. The two-phonon peak for all compounds remains at finite energy below T_{CDW}, in contrast to the complete softening to zero energy for the LA phonon observed in neutron scattering experiments[15]. This is not well understood. A possible explanation is that the two-phonon scattering involves one soft phonon and a normal phonon[16].

Remarkably, the temperature dependent two-phonon peak frequency are highly consistent for $NbSe_2$ samples of differing thickness, as shown in Supplementary Figure 13d. (For the monolayer case we cannot determine the peak frequency below 75 K, most likely due to the influence of the adjacent amplitude mode.) We infer that in $NbSe_2$ samples of all thickness, the LA branch showing the Kohn anomaly softens in the exact same manner upon decreasing temperature, and the CDW wavevector is the same from the bulk to the monolayer, i.e. at $\mathbf{q}_{\mathrm{CDW}} \approx \frac{\mathbf{2}}{\mathbf{3}}\mathbf{\Gamma}\mathrm{M}$. Note that in a first-principles calculation of monolayer $NbSe_2$[17] $\mathbf{q}_{\mathrm{CDW}}$ is predicted to shift to approximately $\frac{1}{2}\Gamma\mathrm{M}$.

For $TaSe_2$ and TaS_2, the two-phonon scattering peak in atomically thin samples are located at similar frequencies as that of the bulk, indicating a similar $\mathbf{q}_{\mathrm{CDW}}$. The temperature dependence of the peak frequency also exhibits a similar trend in samples of all thickness. At low temperature, for most samples except bilayer $TaSe_2$, the mode becomes too weak for a reliable analysis.

In bulk NbS_2, the two-phonon peak at $\sim$250 cm^{-1} redshifts drastically from 300 K to 4 K by almost 52% (cf. 37% in bulk $NbSe_2$). However, a CDW transition is known to be absent. When the sample thickness reduces to bilayer and monolayer, the same two-phonon peak near 250 cm^{-1} remains. However, extra broad peaks appear at lower energy, whose profiles are better fitted with two Gaussian terms. The extracted frequencies for these two new peaks show much less temperature variation than seen for the 250 cm^{-1} mode (Supplementary Figure 13p). Their origin requires further investigation.

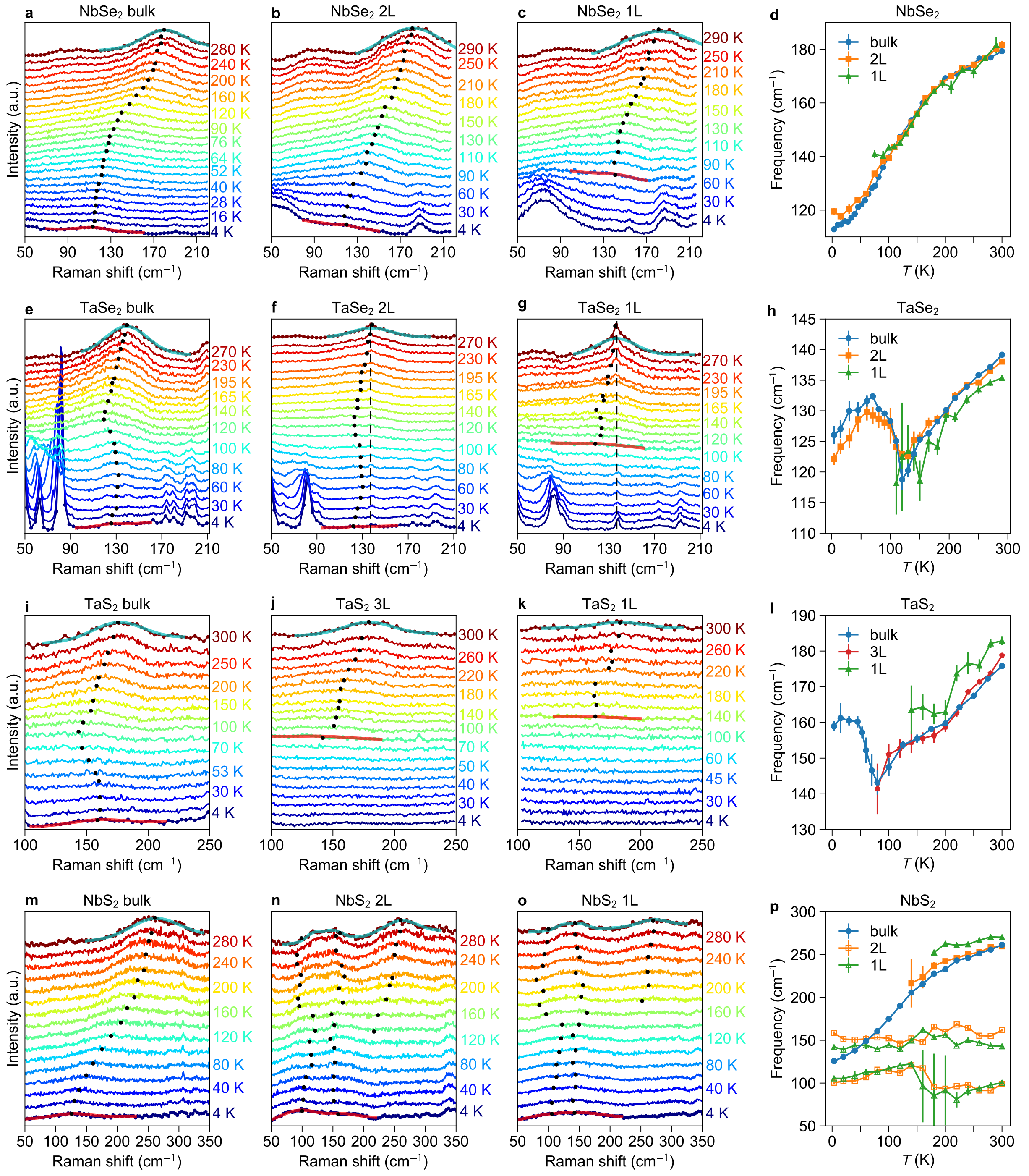

Supplementary Figure 13. **Analysis of the two-phonon scattering peaks. a–c** Raman scattering spectra for $NbSe_2$ corresponding to those in Supplementary Figure 3, vertically shifted for clarity. The spectral range spanning the two-phonon scattering peak is fitted to a model combining a linear background and a Gaussian function. The resultant fits are shown as smooth solid lines for the highest and lowest temperature data suitable for such analysis. The fitted peak frequencies are marked as dots, whose temperature dependence is summarized in **d**. Other rows show the results for $TaSe_2$, TaS_2, and $NbSe_2$. For bilayer and monolayer NbS_2 we included two more Lorentzians in the fits to account for the extra peaks below 200 cm^{-1}. The vertical dashed lines in **f** and **g** indicate a mode absent in the bulk $TaSe_2$. Error bars in the last column represent uncertainties from the fitting analysis.

Supplementary Note 11. Electron-phonon coupling in $NbSe_2$

According to mean-field theory[9,18], the electron-phonon coupling constant λ can be calculated as $\lambda = \omega_A^2/\Omega_0^2$, where ω_A is the amplitude mode frequency in the low-temperature limit and Ω_0 the bare soft mode frequency, respectively. This method was used in Supplementary Reference 19 to obtain the layer-number dependence of λ in $NbSe_2$. Here we adopt the same method for estimating λ in $NbSe_2$. The fitting analysis in Supplementary Note 7 and Supplementary Note 10 yields $\omega_A = 28.0\ \mathrm{cm}^{-1}, 51.1\ \mathrm{cm}^{-1},$ and $73.3\ \mathrm{cm}^{-1}$ at 4 K and $\Omega_0 = 89.7\ \mathrm{cm}^{-1}, 90.8\ \mathrm{cm}^{-1},$ and $90.8\ \mathrm{cm}^{-1}$ as 300 K (i.e. half of the two-phonon scattering peak far above T_{CDW}) for bulk, bilayer, and monolayer samples, respectively. The corresponding values for λ are 0.10, 0.32, 0.65. These results are consistent with those reported in Supplementary Reference 19, as compared in Supplementary Figure 14. The monolayer value is also consistent with that from first-principle calculations ($\lambda = 0.7$) shown in Figure 5 of the main text.

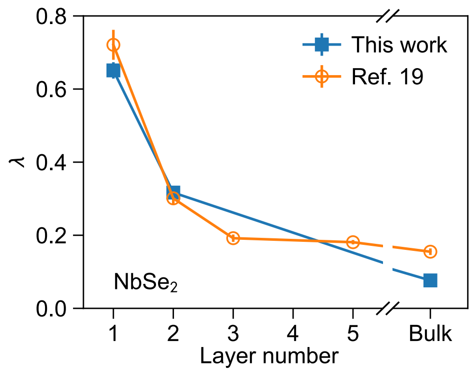

Supplementary Figure 14. **Electron-phonon coupling constant in $NbSe_2$.** Error bars are propagated from those in ω_A and Ω_0. The results in this work and from Supplementary Reference 19 are compared.

Supplementary References

---

1 Lide, D. R. *Handbook of Chemistry and Physics.* 85th ed., pp. 9–76 (CRC Press, Boca Raton, 2004).

2 Hill, H. M. *et al.* Comprehensive optical characterization of atomically thin $NbSe_2$. *Phys. Rev. B* **98**, 165109 (2018).

3 Zhao, Y. *et al.* Interlayer breathing and shear modes in few-trilayer MoS_2 and WSe_2. *Nano Lett.* **19**, 1007–1015 (2013).

4 Menéndez, J. & Cardona, M. Temperature dependence of the first-order Raman scattering by phonons in Si, Ge, and α-Sn: anharmonic effects. *Phys. Rev. B* **29**, 2051–2059 (1984).

5 Lucazeau, G. Effect of pressure and temperature on Raman spectra of solids: anharmonicity. *J. Raman Spectrosc.* **34**, 478–496 (2003).

6 Leroux, M. *et al.* Anharmonic suppression of charge density waves in 2H-NbS_2. *Phys. Rev. B* **86**, 155125 (2012).

7 Heil, C. *et al.* Origin of superconductivity and latent charge density wave in NbS_2. *Phys. Rev. Lett.* **119**, 087003 (2017).

8 Fano, U. Effects of configuration interaction on intensities and phase shifts. *Phys. Rev.* **124**, 1866–1878 (1961).

9 Rice, M. J. & Strässler, S. Theory of the soft phonon mode and dielectric constant below the Peierls transition temperature. *Solid State Commun.* **13**, 1931–1933 (1973).

10 Joshi, J. *et al.* Short-range charge density wave order in 2H-TaS_2. *Phys. Rev. B* **99**, 245144 (2019).

11 Tsang, J. C., Smith, J. E. & Shafer, M. W. Raman spectroscopy of soft modes at the charge-density-wave phase transition in 2H-$NbSe_2$. *Phys. Rev. Lett.* **37**, 1407–1410 (1976).

12 Hill, H. M. *et al.* Phonon origin and lattice evolution in charge density wave states. *Phys. Rev. B* **99**, 174110 (2019).

13 Sugai, S. Generalized electronic susceptibility and charge density waves in transition metal dichalcogenides. *Physica B+C* **117–118**, 587–589 (1983).

14 Klein, M. V. Theory of two-phonon Raman scattering in transition metals and compounds. *Phys. Rev. B* **23**, 4208-4223 (1981).

15 Weber, F. *et al.* Extended phonon collapse and the origin of the charge-density wave in 2H–$NbSe_2$. *Phys. Rev. Lett.* **107**, 107403 (2011).

16 Wu, Y. *et al.* Raman scattering spectra in the normal phase of 2H–$NbSe_2$. *J. Phys. D: Appl. Phys.* **41**, 175408 (2008).

17 Calandra, M., Mazin, I. I.& Mauri, F. Effect of dimensionality on the charge-density wave in few-layer $NbSe_2$. *Phys. Rev. B* **80**, 241108 (2009).

18 Grüner, G. *Density Waves in Solids.* Advanced book program: Addison-Wesley (Perseus Book Group, 2000).

19 Xi, X. *et al.* Strongly enhanced charge-density-wave order in monolayer $NbSe_2$. *Nat. Nanotech.* **10**, 765 (2015).
